# Supplementary material for: Polyketide synthase gene clusters in the ericoid mycorrhizal fungus Oidiodendron maius: genome-wide prediction, expression profiling, and functional characterization of knockout mutants
Source: Front Plant Sci. 2026 Sep 16;17:1924430. doi: 10.3389/fpls.2026.1924430 (PMC13623903; doi:10.3389/fpls.2026.1924430)
Supplement: Supplementary file 1 [file SupplementaryFile1.zip › Supplementary Materials/Supplementary Folder 1.zip!/Oidma1_AssemblyScaffolds_Repeatmasked/knownclusterblast/cluster12/jgi.p_Oidma1_157876_mibig_hits.html]

| MIBiG Protein | Description | MIBiG Cluster | MiBiG Product | % ID | % Coverage | BLAST Score | E-value |
| --- | --- | --- | --- | --- | --- | --- | --- |
| ADY16687.1 | TqaC | BGC0001142 | Tryptoquialanine\_biosynthetic\_gene\_cluster | 32 | 89.7959183673 | 127 | 2.7e-29 |
| CBF73678.1 | conserved\_hypothetical\_protein | BGC0000101 | Monodictyphenone\_biosynthetic\_gene\_cluster | 27 | 66.1807580175 | 63 | 4.8e-10 |
